# Methyl 3-(4-bromo­phen­yl)-1-methyl-1,2,3,3a,4,9b-hexa­hydro­benzo[*f*]chromeno[4,3-*b*]pyrrole-3a-carboxyl­ate

**DOI:** 10.1107/S1600536809029389

**Published:** 2009-07-29

**Authors:** S. Nirmala, E. Theboral Sugi Kamala, L. Sudha, S. Kathiravan, R. Raghunathan

**Affiliations:** a Department of Physics, Easwari Engineering College, Ramapuram, Chennai 600 089, India; b Department of Physics, SRM University, Ramapuram Campus, Chennai 600 089, India; c Department of Organic Chemistry, University of Madras, Guindy Campus, Chennai 600 025, India

## Abstract

In the title compound, C_24_H_22_BrNO_3_, the dihydro­pyran ring adopts a half-chair conformation, whereas the pyrrolidine ring is in an envelope conformation. The bromo­phenyl group is oriented at an angle of 66.44 (4)° with respect to the naphthalene ring system. In the crystal structure, mol­ecules are linked into centrosymmetric dimers by C—H⋯π inter­actions and the dimers are connected *via* C—H⋯Br hydrogen bonds. The crystal structure is further stabilized by π–π inter­actions [centroid–centroid distance = 3.453 (1) Å].

## Related literature

For the biological activity of pyrrole derivatives, see: Biava *et al.* (2005 ▶); Borthwick *et al.* (2000 ▶); Caine (1993 ▶); Carlson (1993 ▶); Fernandes *et al.* (2004 ▶); Jiang *et al.* (2004 ▶); Sokoloff *et al.* (1990 ▶); Tidey (1992 ▶); Wilner (1985 ▶). For a related structure, see: Nirmala *et al.* (2009 ▶). For ring-puckering parameters, see: Cremer & Pople (1975 ▶).
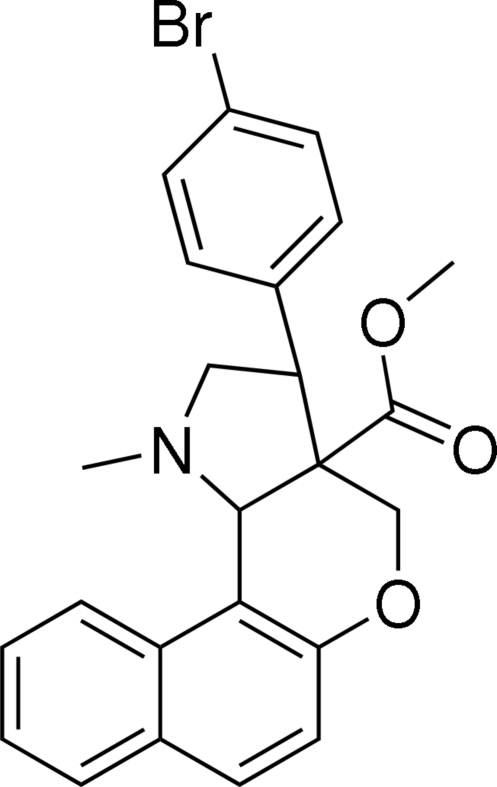


## Experimental

### 

#### Crystal data

C_24_H_22_BrNO_3_
*M* *_r_* = 452.34
Monoclinic, 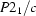

*a* = 12.7856 (4) Å
*b* = 19.9348 (6) Å
*c* = 8.0189 (3) Å
β = 106.163 (2)°
*V* = 1963.06 (11) Å^3^
*Z* = 4
Mo *K*α radiation
μ = 2.12 mm^−1^
*T* = 293 K
0.25 × 0.20 × 0.15 mm

#### Data collection

Bruker Kappa APEXII area-detector diffractometer
Absorption correction: multi-scan (Blessing, 1995 ▶) *T*_min_ = 0.619, *T*_max_ = 0.742
49847 measured reflections
6162 independent reflections
4068 reflections with *I* > 2σ(*I*)
*R*_int_ = 0.031

#### Refinement

*R*[*F* ^2^ > 2σ(*F* ^2^)] = 0.038
*wR*(*F* ^2^) = 0.102
*S* = 1.02
6162 reflections
262 parameters
H-atom parameters constrained
Δρ_max_ = 0.58 e Å^−3^
Δρ_min_ = −0.51 e Å^−3^

### 

Data collection: *APEX2* (Bruker, 2004 ▶); cell refinement: *SAINT* (Bruker, 2004 ▶); data reduction: *SAINT*; program(s) used to solve structure: *SHELXS97* (Sheldrick, 2008 ▶); program(s) used to refine structure: *SHELXL97* (Sheldrick, 2008 ▶); molecular graphics: *ORTEP-3* (Farrugia, 1997 ▶); software used to prepare material for publication: *PLATON* (Spek, 2009 ▶).

## Supplementary Material

Crystal structure: contains datablocks I, global. DOI: 10.1107/S1600536809029389/ci2858sup1.cif

Structure factors: contains datablocks I. DOI: 10.1107/S1600536809029389/ci2858Isup2.hkl

Additional supplementary materials:  crystallographic information; 3D view; checkCIF report

## Figures and Tables

**Table 1:** Hydrogen-bond geometry (Å, °)

*D*—H⋯*A*	*D*—H	H⋯*A*	*D*⋯*A*	*D*—H⋯*A*
C24—H24*A*⋯Br1^i^	0.96	2.84	3.789 (3)	172
C20—H20⋯*Cg*1^ii^	0.93	2.77	3.653 (2)	160

Symmetry codes: (i) 
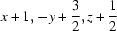
; (ii) 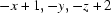. *Cg*1 is the centroid of the C3–C8 ring.

**Fig. 1.**
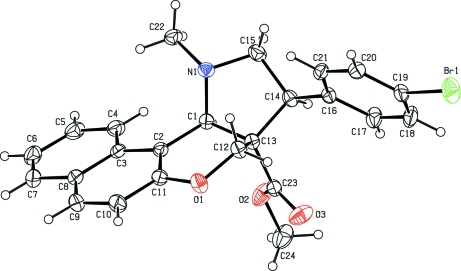
The molecular structure of the title compound, with 30% probability displacement ellipsoids.

**Fig. 2.**
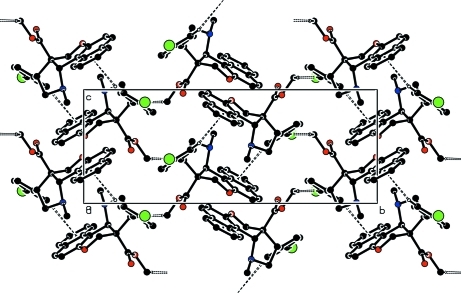
The packing of the molecules viewed down a axis. H atoms not involed in C—H···Br and C—H···π interactions have been omitted.

## supplementary crystallographic information

### Comment

Chromenopyrrole compounds are used in the treatment of impulsive disorders (Caine, 1993), aggressiveness (Tidey, 1992), parkinson's disease (Carlson, 1993), psychoses, memory disorders (Sokoloff *et al.*, 1990), anxiety and depression (Wilner, 1985). Pyrrole derivatives have good *in vitro* activities against mycobacteria and candidae (Biava *et al.*, 2005). These derivatives also possess anti-inflammatory (Fernandes *et al.*, 2004) and antiviral (Borthwick *et al.*, 2000) activities. It has also been shown that N-substituted pyrrole derivatives inhibit human immuno deficiency virus type-I (HIV-I) (Jiang *et al.*, 2004). In view of its medicinal importance, the crystal structure determination of the title compound was undertaken.

The geometric parameters of the title molecule (Fig. 1) agree well with those reported for a similar structure (Nirmala *et al.*, 2009). The sum of bond angles around atom N1 [332.0 (8)°] is in accordance with *sp*^3^ hybridization. The naphthalene ring system (C2-C11) and the bromophenyl group (Br1/C16-C21) are oriented at an angle of 66.44 (4)° with respect to each other. The heterocyclic ring (O1/C1/C2/C11-C13) of the chromenopyrrole unit adopts a half chair conformation with puckering parameters *Q* = 0.468 (2) Å, *θ* = 132.5 (2)° and *φ* = 83.1 (3)° (Cremer & Pople, 1975). The pyrrolidine ring (N1/C1/C13-C15) adopts an envelope conformation with puckering parameters *q*_2_ = 0.433 (2) Å and *φ* = 214.5 (3)° (Cremer & Pople, 1975). Atom C1 deviates by -0.654 Å from the least-square plane through the remaining four atoms.

The crystal packing is stabilized by intermolecular C—H···Br hydrogen bonds. The molecules are linked into centrosymmetric dimers by C—H···π (C20—H20···*Cg1*; *Cg1* is the centroid of the C3—C8 ring) interactions (Table 1). In addition, π–π interactions between C3—C8 rings at (*x*, *y*, *z*) and (2 -*x*, 1 -*y*, 2 -*z*) stabilize the structure, with a centroid-to-centroid distance of 3.453 (1) Å.

### Experimental

A mixture of (*Z*)-methyl 2-((1-formylnaphthalen-2-yloxy)methyl)-3-(4-bromophenyl)acrylate (20 mmol) and sarcosine (30 mmol) were refluxed in benzene for 20 h and the solvent was removed under reduced pressure. The crude product was subjected to column chromatography to get the pure product. Chloroform and methanol (1:1) solvent mixture was used for the crystallization under slow evaporation method.

### Refinement

H atoms were placed in idealized positions and allowed to ride on their parent atoms, with C-H = 0.93, 0.98, 0.97 and 0.96 Å for aromatic, methine, methylene and methyl H respectively, and *U*_iso_(H) = 1.5U_eq_(C) for methyl H and *U*_iso_(H) = 1.2U_eq_(C) for all other H atoms. Reflections 110 and 100 were omitted during the final cycles of refinement since they were seriously effected by the beamstop.

### Crystal data

**Table:**

C_24_H_22_BrNO_3_	*F*(000) = 928
*M**_r_* = 452.34	*D*_x_ = 1.531 Mg m^−^^3^
Monoclinic, *P*2_1_/*c*	Mo *K*α radiation, λ = 0.71073 Å
Hall symbol: -P 2ybc	Cell parameters from 14860 reflections
*a* = 12.7856 (4) Å	θ = 2.6–25.2°
*b* = 19.9348 (6) Å	µ = 2.12 mm^−^^1^
*c* = 8.0189 (3) Å	*T* = 293 K
β = 106.163 (2)°	Prism, colourless
*V* = 1963.06 (11) Å^3^	0.25 × 0.20 × 0.15 mm
*Z* = 4	

### Data collection

**Table:**

Bruker Kappa APEXII area-detector diffractometer	6162 independent reflections
Radiation source: fine-focus sealed tube	4068 reflections with *I* > 2σ(*I*)
graphite	*R*_int_ = 0.031
ω and φ scans	θ_max_ = 30.9°, θ_min_ = 2.0°
Absorption correction: multi-scan (Blessing, 1995)	*h* = −18→18
*T*_min_ = 0.619, *T*_max_ = 0.742	*k* = −16→28
49847 measured reflections	*l* = −11→11

### Refinement

**Table:**

Refinement on *F*^2^	Primary atom site location: structure-invariant direct methods
Least-squares matrix: full	Secondary atom site location: difference Fourier map
*R*[*F*^2^ > 2σ(*F*^2^)] = 0.038	Hydrogen site location: inferred from neighbouring sites
*wR*(*F*^2^) = 0.102	H-atom parameters constrained
*S* = 1.02	*w* = 1/[σ^2^(*F*_o_^2^) + (0.0439*P*)^2^ + 0.7192*P*] where *P* = (*F*_o_^2^ + 2*F*_c_^2^)/3
6162 reflections	(Δ/σ)_max_ = 0.001
262 parameters	Δρ_max_ = 0.58 e Å^−^^3^
0 restraints	Δρ_min_ = −0.51 e Å^−^^3^

### Special details

**Table:**

Geometry. All e.s.d.'s (except the e.s.d. in the dihedral angle between two l.s. planes) are estimated using the full covariance matrix. The cell e.s.d.'s are taken into account individually in the estimation of e.s.d.'s in distances, angles and torsion angles; correlations between e.s.d.'s in cell parameters are only used when they are defined by crystal symmetry. An approximate (isotropic) treatment of cell e.s.d.'s is used for estimating e.s.d.'s involving l.s. planes.
Refinement. Refinement of *F*^2^ against ALL reflections. The weighted *R*-factor *wR* and goodness of fit *S* are based on *F*^2^, conventional *R*-factors *R* are based on *F*, with *F* set to zero for negative *F*^2^. The threshold expression of *F*^2^ > σ(*F*^2^) is used only for calculating *R*-factors(gt) *etc*. and is not relevant to the choice of reflections for refinement. *R*-factors based on *F*^2^ are statistically about twice as large as those based on *F*, and *R*- factors based on ALL data will be even larger.

### Fractional atomic coordinates and isotropic or equivalent isotropic displacement parameters (Å^2^)

**Table:**

	*x*	*y*	*z*	*U*_iso_*/*U*_eq_	
C1	0.68758 (13)	0.58289 (8)	0.6893 (2)	0.0281 (3)	
H1	0.7433	0.6171	0.6940	0.034*	
C2	0.73957 (13)	0.52048 (8)	0.7836 (2)	0.0287 (3)	
C3	0.84557 (13)	0.49812 (8)	0.7827 (2)	0.0306 (3)	
C4	0.91361 (15)	0.53431 (10)	0.7029 (2)	0.0381 (4)	
H4	0.8889	0.5745	0.6470	0.046*	
C5	1.01452 (16)	0.51164 (11)	0.7061 (2)	0.0455 (5)	
H5	1.0576	0.5367	0.6530	0.055*	
C6	1.05416 (17)	0.45149 (12)	0.7878 (3)	0.0510 (5)	
H6	1.1226	0.4360	0.7869	0.061*	
C7	0.99246 (17)	0.41579 (11)	0.8685 (3)	0.0474 (5)	
H7	1.0195	0.3759	0.9241	0.057*	
C8	0.88813 (15)	0.43783 (9)	0.8699 (2)	0.0357 (4)	
C9	0.82649 (16)	0.40202 (9)	0.9600 (3)	0.0425 (4)	
H9	0.8535	0.3620	1.0149	0.051*	
C10	0.72848 (16)	0.42484 (9)	0.9681 (2)	0.0417 (4)	
H10	0.6893	0.4013	1.0309	0.050*	
C11	0.68588 (14)	0.48451 (8)	0.8811 (2)	0.0329 (3)	
C12	0.52824 (14)	0.55294 (8)	0.7923 (2)	0.0338 (4)	
H12A	0.4714	0.5700	0.8396	0.041*	
H12B	0.4935	0.5324	0.6810	0.041*	
C13	0.59903 (13)	0.61089 (8)	0.7656 (2)	0.0289 (3)	
C14	0.53395 (14)	0.65915 (8)	0.6186 (2)	0.0333 (4)	
H14	0.5695	0.7031	0.6403	0.040*	
C15	0.55720 (16)	0.63062 (10)	0.4547 (2)	0.0424 (4)	
H15A	0.4896	0.6189	0.3692	0.051*	
H15B	0.5952	0.6635	0.4040	0.051*	
C16	0.41597 (13)	0.66967 (8)	0.6127 (2)	0.0308 (3)	
C17	0.38794 (16)	0.72456 (9)	0.6967 (3)	0.0449 (5)	
H17	0.4420	0.7544	0.7536	0.054*	
C18	0.28173 (17)	0.73619 (10)	0.6981 (3)	0.0491 (5)	
H18	0.2645	0.7732	0.7558	0.059*	
C19	0.20242 (15)	0.69243 (9)	0.6136 (2)	0.0379 (4)	
C20	0.22678 (14)	0.63726 (9)	0.5282 (2)	0.0362 (4)	
H20	0.1721	0.6077	0.4715	0.043*	
C21	0.33319 (14)	0.62635 (8)	0.5280 (2)	0.0337 (4)	
H21	0.3499	0.5892	0.4699	0.040*	
C22	0.68903 (16)	0.55698 (9)	0.3883 (2)	0.0385 (4)	
H22A	0.7326	0.5177	0.4259	0.058*	
H22B	0.7357	0.5945	0.3858	0.058*	
H22C	0.6413	0.5496	0.2740	0.058*	
C23	0.63964 (14)	0.65052 (8)	0.9323 (2)	0.0338 (4)	
C24	0.7836 (2)	0.71536 (12)	1.1053 (3)	0.0631 (7)	
H24A	0.8555	0.7299	1.1072	0.095*	
H24B	0.7869	0.6896	1.2078	0.095*	
H24C	0.7378	0.7538	1.1018	0.095*	
N1	0.62481 (12)	0.57086 (7)	0.50776 (18)	0.0322 (3)	
O1	0.58933 (10)	0.50339 (6)	0.90663 (17)	0.0412 (3)	
O2	0.73947 (11)	0.67466 (8)	0.9542 (2)	0.0529 (4)	
O3	0.58771 (13)	0.66160 (9)	1.03113 (19)	0.0594 (4)	
Br1	0.056104 (18)	0.708461 (13)	0.61041 (4)	0.06427 (10)	

### Atomic displacement parameters (Å^2^)

**Table:**

	*U*^11^	*U*^22^	*U*^33^	*U*^12^	*U*^13^	*U*^23^
C1	0.0292 (8)	0.0257 (7)	0.0314 (8)	−0.0020 (6)	0.0121 (6)	0.0029 (6)
C2	0.0324 (8)	0.0275 (7)	0.0275 (8)	0.0002 (6)	0.0103 (7)	0.0006 (6)
C3	0.0332 (8)	0.0333 (8)	0.0255 (8)	0.0009 (6)	0.0085 (6)	−0.0059 (6)
C4	0.0382 (9)	0.0452 (10)	0.0322 (9)	0.0007 (7)	0.0118 (7)	−0.0013 (7)
C5	0.0370 (10)	0.0638 (13)	0.0382 (10)	−0.0007 (9)	0.0144 (8)	−0.0083 (9)
C6	0.0379 (10)	0.0685 (14)	0.0469 (12)	0.0140 (10)	0.0121 (9)	−0.0119 (10)
C7	0.0477 (11)	0.0486 (11)	0.0429 (11)	0.0169 (9)	0.0076 (9)	−0.0056 (9)
C8	0.0382 (9)	0.0375 (9)	0.0297 (9)	0.0063 (7)	0.0064 (7)	−0.0052 (7)
C9	0.0523 (11)	0.0324 (9)	0.0405 (10)	0.0103 (8)	0.0092 (9)	0.0053 (8)
C10	0.0512 (11)	0.0344 (9)	0.0426 (10)	0.0008 (8)	0.0181 (9)	0.0105 (8)
C11	0.0362 (9)	0.0315 (8)	0.0326 (9)	0.0004 (7)	0.0124 (7)	0.0030 (7)
C12	0.0304 (8)	0.0343 (8)	0.0397 (9)	0.0005 (6)	0.0146 (7)	0.0089 (7)
C13	0.0278 (8)	0.0288 (7)	0.0324 (8)	0.0006 (6)	0.0122 (6)	0.0043 (6)
C14	0.0322 (8)	0.0302 (8)	0.0375 (9)	0.0009 (6)	0.0100 (7)	0.0072 (7)
C15	0.0399 (10)	0.0523 (11)	0.0375 (10)	0.0099 (8)	0.0150 (8)	0.0143 (8)
C16	0.0321 (8)	0.0260 (7)	0.0334 (9)	0.0029 (6)	0.0077 (7)	0.0028 (6)
C17	0.0396 (10)	0.0328 (9)	0.0559 (12)	0.0008 (7)	0.0024 (9)	−0.0141 (8)
C18	0.0480 (11)	0.0385 (10)	0.0585 (13)	0.0110 (8)	0.0106 (10)	−0.0155 (9)
C19	0.0348 (9)	0.0392 (9)	0.0401 (10)	0.0095 (7)	0.0110 (8)	0.0041 (7)
C20	0.0348 (9)	0.0346 (9)	0.0373 (9)	−0.0027 (7)	0.0068 (7)	−0.0015 (7)
C21	0.0377 (9)	0.0293 (8)	0.0346 (9)	0.0016 (7)	0.0110 (7)	−0.0045 (7)
C22	0.0453 (10)	0.0421 (10)	0.0315 (9)	−0.0002 (8)	0.0164 (8)	0.0016 (7)
C23	0.0333 (9)	0.0317 (8)	0.0367 (9)	0.0059 (7)	0.0103 (7)	0.0042 (7)
C24	0.0480 (13)	0.0636 (14)	0.0739 (16)	−0.0068 (10)	0.0107 (11)	−0.0321 (12)
N1	0.0346 (7)	0.0358 (7)	0.0280 (7)	−0.0006 (6)	0.0118 (6)	0.0051 (6)
O1	0.0393 (7)	0.0416 (7)	0.0498 (8)	0.0059 (5)	0.0238 (6)	0.0190 (6)
O2	0.0394 (7)	0.0582 (9)	0.0646 (9)	−0.0116 (6)	0.0203 (7)	−0.0283 (7)
O3	0.0555 (9)	0.0822 (11)	0.0476 (8)	−0.0085 (8)	0.0263 (7)	−0.0169 (8)
Br1	0.04256 (13)	0.07000 (17)	0.0859 (2)	0.01747 (10)	0.02724 (12)	0.00945 (12)

### Geometric parameters (Å, °)

**Table:**

C1—N1	1.473 (2)	C14—C16	1.511 (2)
C1—C2	1.510 (2)	C14—C15	1.536 (3)
C1—C13	1.534 (2)	C14—H14	0.98
C1—H1	0.98	C15—N1	1.464 (2)
C2—C11	1.377 (2)	C15—H15A	0.97
C2—C3	1.429 (2)	C15—H15B	0.97
C3—C4	1.413 (2)	C16—C17	1.383 (2)
C3—C8	1.421 (2)	C16—C21	1.388 (2)
C4—C5	1.361 (3)	C17—C18	1.381 (3)
C4—H4	0.93	C17—H17	0.93
C5—C6	1.392 (3)	C18—C19	1.366 (3)
C5—H5	0.93	C18—H18	0.93
C6—C7	1.353 (3)	C19—C20	1.376 (3)
C6—H6	0.93	C19—Br1	1.8910 (18)
C7—C8	1.408 (3)	C20—C21	1.378 (2)
C7—H7	0.93	C20—H20	0.93
C8—C9	1.403 (3)	C21—H21	0.93
C9—C10	1.352 (3)	C22—N1	1.452 (2)
C9—H9	0.93	C22—H22A	0.96
C10—C11	1.410 (2)	C22—H22B	0.96
C10—H10	0.93	C22—H22C	0.96
C11—O1	1.360 (2)	C23—O3	1.188 (2)
C12—O1	1.424 (2)	C23—O2	1.329 (2)
C12—C13	1.519 (2)	C24—O2	1.437 (3)
C12—H12A	0.97	C24—H24A	0.96
C12—H12B	0.97	C24—H24B	0.96
C13—C23	1.514 (2)	C24—H24C	0.96
C13—C14	1.569 (2)		
			
N1—C1—C2	113.78 (13)	C16—C14—C13	115.15 (13)
N1—C1—C13	101.17 (13)	C15—C14—C13	103.15 (13)
C2—C1—C13	111.80 (13)	C16—C14—H14	107.0
N1—C1—H1	109.9	C15—C14—H14	107.0
C2—C1—H1	109.9	C13—C14—H14	107.0
C13—C1—H1	109.9	N1—C15—C14	106.96 (14)
C11—C2—C3	117.65 (15)	N1—C15—H15A	110.3
C11—C2—C1	119.57 (14)	C14—C15—H15A	110.3
C3—C2—C1	122.72 (14)	N1—C15—H15B	110.3
C4—C3—C8	117.08 (16)	C14—C15—H15B	110.3
C4—C3—C2	123.20 (15)	H15A—C15—H15B	108.6
C8—C3—C2	119.69 (15)	C17—C16—C21	117.69 (16)
C5—C4—C3	121.46 (18)	C17—C16—C14	119.11 (15)
C5—C4—H4	119.3	C21—C16—C14	123.20 (15)
C3—C4—H4	119.3	C18—C17—C16	121.72 (17)
C4—C5—C6	120.93 (19)	C18—C17—H17	119.1
C4—C5—H5	119.5	C16—C17—H17	119.1
C6—C5—H5	119.5	C19—C18—C17	118.97 (17)
C7—C6—C5	119.57 (18)	C19—C18—H18	120.5
C7—C6—H6	120.2	C17—C18—H18	120.5
C5—C6—H6	120.2	C18—C19—C20	121.18 (17)
C6—C7—C8	121.30 (19)	C18—C19—Br1	119.58 (14)
C6—C7—H7	119.4	C20—C19—Br1	119.23 (14)
C8—C7—H7	119.4	C19—C20—C21	119.14 (16)
C9—C8—C7	120.97 (17)	C19—C20—H20	120.4
C9—C8—C3	119.39 (16)	C21—C20—H20	120.4
C7—C8—C3	119.61 (18)	C20—C21—C16	121.29 (15)
C10—C9—C8	120.98 (17)	C20—C21—H21	119.4
C10—C9—H9	119.5	C16—C21—H21	119.4
C8—C9—H9	119.5	N1—C22—H22A	109.5
C9—C10—C11	119.71 (17)	N1—C22—H22B	109.5
C9—C10—H10	120.1	H22A—C22—H22B	109.5
C11—C10—H10	120.1	N1—C22—H22C	109.5
O1—C11—C2	123.97 (15)	H22A—C22—H22C	109.5
O1—C11—C10	113.62 (15)	H22B—C22—H22C	109.5
C2—C11—C10	122.39 (16)	O3—C23—O2	122.60 (17)
O1—C12—C13	112.14 (14)	O3—C23—C13	124.61 (16)
O1—C12—H12A	109.2	O2—C23—C13	112.73 (14)
C13—C12—H12A	109.2	O2—C24—H24A	109.5
O1—C12—H12B	109.2	O2—C24—H24B	109.5
C13—C12—H12B	109.2	H24A—C24—H24B	109.5
H12A—C12—H12B	107.9	O2—C24—H24C	109.5
C23—C13—C12	110.09 (14)	H24A—C24—H24C	109.5
C23—C13—C1	115.59 (13)	H24B—C24—H24C	109.5
C12—C13—C1	108.33 (13)	C22—N1—C15	111.10 (13)
C23—C13—C14	108.83 (13)	C22—N1—C1	115.52 (14)
C12—C13—C14	111.11 (13)	C15—N1—C1	105.67 (13)
C1—C13—C14	102.70 (13)	C11—O1—C12	116.96 (12)
C16—C14—C15	117.01 (15)	C23—O2—C24	116.95 (16)
			
N1—C1—C2—C11	−94.11 (18)	C12—C13—C14—C16	37.3 (2)
C13—C1—C2—C11	19.7 (2)	C1—C13—C14—C16	152.89 (14)
N1—C1—C2—C3	88.72 (18)	C23—C13—C14—C15	147.21 (14)
C13—C1—C2—C3	−157.44 (15)	C12—C13—C14—C15	−91.41 (16)
C11—C2—C3—C4	−173.26 (16)	C1—C13—C14—C15	24.21 (16)
C1—C2—C3—C4	4.0 (2)	C16—C14—C15—N1	−125.38 (15)
C11—C2—C3—C8	4.7 (2)	C13—C14—C15—N1	2.14 (18)
C1—C2—C3—C8	−178.13 (15)	C15—C14—C16—C17	−143.63 (18)
C8—C3—C4—C5	1.5 (3)	C13—C14—C16—C17	94.9 (2)
C2—C3—C4—C5	179.42 (16)	C15—C14—C16—C21	37.0 (2)
C3—C4—C5—C6	0.3 (3)	C13—C14—C16—C21	−84.4 (2)
C4—C5—C6—C7	−1.5 (3)	C21—C16—C17—C18	0.5 (3)
C5—C6—C7—C8	0.7 (3)	C14—C16—C17—C18	−178.94 (19)
C6—C7—C8—C9	−177.30 (18)	C16—C17—C18—C19	−0.3 (3)
C6—C7—C8—C3	1.1 (3)	C17—C18—C19—C20	0.2 (3)
C4—C3—C8—C9	176.27 (16)	C17—C18—C19—Br1	−178.80 (16)
C2—C3—C8—C9	−1.8 (2)	C18—C19—C20—C21	−0.2 (3)
C4—C3—C8—C7	−2.1 (2)	Br1—C19—C20—C21	178.80 (13)
C2—C3—C8—C7	179.83 (16)	C19—C20—C21—C16	0.3 (3)
C7—C8—C9—C10	176.89 (18)	C17—C16—C21—C20	−0.5 (3)
C3—C8—C9—C10	−1.5 (3)	C14—C16—C21—C20	178.91 (16)
C8—C9—C10—C11	1.8 (3)	C12—C13—C23—O3	−37.5 (2)
C3—C2—C11—O1	173.77 (15)	C1—C13—C23—O3	−160.58 (17)
C1—C2—C11—O1	−3.5 (3)	C14—C13—C23—O3	84.5 (2)
C3—C2—C11—C10	−4.5 (3)	C12—C13—C23—O2	145.27 (15)
C1—C2—C11—C10	178.16 (16)	C1—C13—C23—O2	22.1 (2)
C9—C10—C11—O1	−177.08 (17)	C14—C13—C23—O2	−92.74 (17)
C9—C10—C11—C2	1.4 (3)	C14—C15—N1—C22	−155.30 (15)
O1—C12—C13—C23	−67.90 (18)	C14—C15—N1—C1	−29.30 (18)
O1—C12—C13—C1	59.38 (18)	C2—C1—N1—C22	−72.26 (17)
O1—C12—C13—C14	171.46 (13)	C13—C1—N1—C22	167.70 (13)
N1—C1—C13—C23	−160.07 (13)	C2—C1—N1—C15	164.50 (13)
C2—C1—C13—C23	78.49 (17)	C13—C1—N1—C15	44.46 (15)
N1—C1—C13—C12	75.89 (15)	C2—C11—O1—C12	16.4 (2)
C2—C1—C13—C12	−45.55 (18)	C10—C11—O1—C12	−165.22 (16)
N1—C1—C13—C14	−41.73 (14)	C13—C12—O1—C11	−45.0 (2)
C2—C1—C13—C14	−163.17 (13)	O3—C23—O2—C24	0.2 (3)
C23—C13—C14—C16	−84.11 (17)	C13—C23—O2—C24	177.54 (17)

### Hydrogen-bond geometry (Å, °)

**Table:**

*D*—H···*A*	*D*—H	H···*A*	*D*···*A*	*D*—H···*A*
C24—H24A···Br1^i^	0.96	2.84	3.789 (3)	172
C20—H20···Cg1^ii^	0.93	2.77	3.653 (2)	160

Symmetry codes: (i) *x*+1, −*y*+3/2, *z*+1/2; (ii) −*x*+1, −*y*, −*z*+2.
